# A CAVE Survey for Measuring Mathematics Attitudes Based on the Characteristics of Students in Mainland China

**DOI:** 10.3390/bs15040412

**Published:** 2025-03-24

**Authors:** Jing Lv, Ke Wang, Fei Chen, Ali Bicer

**Affiliations:** 1 Department of Psychology, Fudan University, Shanghai 200433, China; 2 Department of Teacher Education, Nicholls State University, Thibodaux, LA 70310, USA; 3 School of Mathematics and Science, Changsha Normal University, Changsha 410100, China; 4 Department of Teaching, Learning, & Culture, Texas A&M University, College Station, TX 77843, USA

**Keywords:** assessments, attitudes toward mathematics, high school students, mainland China, validity

## Abstract

As mathematics is the foundation for pursuing science, technology, engineering, and mathematics (STEM) majors, investigating the assessment of attitudes toward mathematics is essential for promoting the development of the STEM field. However, the work focusing on developing a survey of attitudes toward mathematics is limited in mainland China. This study built a theoretical construct of students’ attitudes toward mathematics based on the characteristics of the educational system in mainland China. A survey researchers could use to measure mainland Chinese students’ attitudes toward mathematics was created and developed according to the proposed theoretical construct. The target survey was validated using exploratory factor analysis (EFA) and evaluated using confirmatory factor analysis (CFA) based on 700 and 778 high school students from mainland China, respectively. Furthermore, the relationship between math attitudes and performance was used to check the construct of the developed survey. The finalized survey includes four scales: confidence (i.e., α = 0.90 with 10 items), anxiety (i.e., α = 0.90 with 10 items), value (i.e., α = 0.70 with 6 items), and enjoyment (i.e., α = 0.71 with 8 items). The overall results show that the survey is reliable and valid to measure secondary students’ attitudes toward mathematics, and thus suggest future studies about measuring Chinese secondary students’ attitudes towards mathematics by employing this survey. This study might draw the attention of researchers to investigate attitudes toward mathematics while exploring their affective and cognitive outcomes related to mathematics.

## 1. Introduction

Mathematics serves as the foundation for the development of STEM (i.e., science, technology, engineering, and mathematics) education. It can influence the understanding of the ideas and concepts of other STEM disciplines (Fitzallen, 2015). Furthermore, the ability to work with data and uncertainty is essential for making informed decisions with ethical, economic, and environmental considerations in mind (English, 2016). As STEM has been the foundation for discovery and technological innovation in the 21st century (Committee on STEM Education, 2018) and strong mathematical literacy paves the way for students to pursue STEM majors (Wang, 2013), students with negative attitudes toward mathematics are less likely to pursue a STEM major (Ramirez et al., 2013). Therefore, to foster scientific and technical talent and prepare the future STEM workforce, educators should focus on sparking students’ interest in mathematics and helping them establish a solid foundation in the subject to instill confidence for pursuing STEM majors in the future.

Unfortunately, mathematics is often considered as a less popular subject (Bragg, 2007; Tseng et al., 2013) due to students’ complicated attitudes toward it. That is, mathematics learning is a difficult process for some students, so they exhibit a decreased level of participation and lesson avoidance. With the increase in grade levels, mathematical problems become more complex, resulting in more students joining the group with negative attitudes toward mathematics (Maloney & Beilock, 2012). On the other hand, mathematics is simply unappealing or socially unacceptable (e.g., females may be discouraged from learning mathematics) for students who may have high aptitude but exhibit low motivation to learn mathematics. As positive attitudes toward mathematics play a key role in promoting mathematics learning (Wang, 2013), mathematics anxiety could make a negative effect on students’ achievement (Bicer et al., 2020b; Vukovic et al., 2013).

To improve students’ mathematical achievement, many studies (e.g., Berger et al., 2020; Kiwanuka et al., 2020) have focused on students’ attitudes toward mathematics and the relationship between these attitudes and achievement. The studies of attitudes toward mathematics include different constructs of enjoyment, motivation, values, confidence, and anxiety (e.g., Laranang & Bondoc, 2020; Živković et al., 2023). To accurately assess attitudes toward mathematics, researchers developed different proper attitude scales based on the teaching situations in their countries or regions such as the US, the UK, Iran, Singapore, Taiwan, and Hong Kong. However, typical scales that measure various constructions of attitudes toward mathematics have rarely been tested with students from mainland China (Zhang et al., 2021), especially, high school students. As China has a quite different culture from other countries, mainland Chinese students may have very different characteristics from non-mainland Chinese students (Brown & Wang, 2016; Chen & Ye, 2024; Lu et al., 2008; Rudowicz & Yue, 2000; Zhang et al., 2016). Although the majority of people in mainland China, Hong Kong, and Taiwan have similar Confucian culture and social practices, they are not a homogeneous Chinese group (Rudowicz & Yue, 2000). As Zhang et al. (2016) described:

What makes the issue of differentiating the Chinese Mainland, Hong Kong, and Taiwan of particular interest is that these regions have undergone different historical developments in the past century and were influenced by different cultural values, resulting in different educational systems in each of these regions. (p. 908).

Although mainland Chinese students have great performance in the Programme for International Student Assessment (PISA) 2012, 2015, and 2018, the question of whether they have the same high positive attitudes toward mathematics as their top-level performance has remained. For example, Leung (2001) claimed that East Asian students’ high achievement results from teachers’ emphasis on developing mathematical fluency rather than rote procedure learning. Leung (2006) further argued that the level of their positive attitudes toward mathematics and mathematical learning did not seem to match their high level of performance in the international comparison. To understand Chinese students’ attitudes toward mathematics deeply, it is essential to assess mainland Chinese students’ attitudes toward mathematics with a survey that has good psychometric properties.

Therefore, this study aims to develop and assess a revised inventory of attitudes toward mathematics based on samples of mainland Chinese high school students. Although this study used data from Chinese students, it can provide a perspective for international readers to understand their students’ attitudes toward math by comparing this survey. Also, this study might help international readers understand possible real reasons for Chinese students’ high performance in international tests (i.e., PISA, TIMSS). Furthermore, this research contributes to the broader discourse on how cultural and linguistic factors influence mathematical learning. International scholars can use these findings to design more effective, inclusive, and culturally responsive educational practices that support diverse student populations.

In general, the current study addresses the following three questions: (1) What specific components are included in the developed survey? (2) Are the psychometric properties of the developed survey acceptable? To understand the appropriateness of the content of each item based on the created or developed construction of students’ attitudes toward mathematics, we address the question (3) of whether the components of the developed survey are significant for math performance.

## 2. Literature Review

### 2.1. Instruments to Assess Attitudes Toward Mathematics

As socio-cognitive theories have suggested that students’ beliefs and expectations are major determinants of their pursuit of and engagement in mathematics courses (e.g., Crombie et al., 2005; Wang, 2013), success in mathematics is greatly determined by personal attitudes. However, there is no consensus on the definition of attitudes toward mathematics due to the differences among sample backgrounds and cultures. Thus, different scales of attitudes toward mathematics from different educational systems (e.g., Aiken, 1974, 1979; Lim & Chapman, 2013; Lin & Huang, 2016; Tapia & Marsh, 2004) have existed in the literature.

Some studies have conceptualized and measured attitudes toward mathematics as multidimensional constructs. In the beginning, Fedon (1958) defined attitudes in two dimensions with like or dislike of mathematics. Later, researchers (e.g., Aiken, 1974) found a significant relationship between attitudes and anxiety in mathematics learning, and attitudes were then defined as a more complex composition with more dimensions, such as anxiety, enjoyment, and value. For example, Aiken (1974) developed a two-factor scale for attitudes toward mathematics: enjoyment and value of mathematics. Other researchers further perfected the definition of attitudes with multiple components and created more scales. One of the most popular instruments is the Fennema–Sherman Mathematics Attitude Scales (FSMAS; Fennema & Sherman, 1976). FSMAS includes nine scales with 108 items: (a) the attitude towards success in mathematics scale, (b) the mathematics as a male domain scale, (c) the mother scale, (d) the father scale, (e) the teacher scale, (f) the confidence in learning mathematics scale, (g) the mathematics anxiety scale, (h) the effectance motivation scale in mathematics, and (i) the mathematics usefulness scale. Although the FSMAS could be used to assess more dimensions of attitudes, it has two major limitations: (a) the number of items is too large for students to pay attention within 20 min (Bradbury, 2016), and (b) it might not be suitable for non-secondary students. Hence, the FSMAS has been modified and applied in different cultures and students with varying grade levels. For example, Betz (1978) employed the scale of math anxiety to examine 652 college students’ mathematics anxiety. Mulhern and Rae (1998) checked the FSMAS using a sample of 196 secondary students from Ireland and developed a shortened version of six separate factors of attitudes toward mathematics including 51 items. Ren et al. (2016) examined lower-primary US teachers’ attitudes toward mathematics by revising and using three scales from FSMAS: confidence, effectance motivation, and anxiety.

Another popular instrument is the Attitudes Toward Mathematics Inventory (ATMI; Tapia & Marsh, 2004). Tapia and Marsh (2004) defined attitudes toward mathematics as a person’s feelings and emotions by constructing four components: value (i.e., the beliefs about the usefulness, relevance, and worth of mathematics in people’s lives), self-confidence (i.e., confidence in mathematics performance), enjoyment (i.e., the degree to enjoy mathematics and mathematics classes), and motivation (i.e., the interest in mathematics and desire to pursue studies in mathematics). Tapia and Marsh (2004) then developed the ATMI by measuring 545 high school students’ attitudes toward mathematics with 40 items.

To assess the ATMI with eastern samples, Lim and Chapman (2013) developed a short version of the ATMI based on Singapore students, including four scales: enjoyment of mathematics (five items), motivation to do mathematics (four items), self-confidence in mathematics (five items), and perceived value of mathematics (five items). Similarly, Lin and Huang (2016) defined attitudes toward mathematics as one’s feelings about mathematics based on his/her beliefs about mathematics. Then, Lin and Huang (2016) developed a Chinese version of the short ATMI based on Taiwan college students including four scales: enjoyment (three items), motivation (three items), self-confidence (four items), and value (four items). However, after using a short version of ATMI in the studies of Lim and Chapman (2013), Lin and Huang (2016) indicated the huge difference between the Asian and Western samples, as well as the noticeable effects of samples on scale development.

Moreover, this kind of study focusing on student samples of mainland China, a special developing country with a complex cultural background, is very limited. Unlike other samples, mainland Chinese students, especially high school students, have higher math anxiety problems due to the common comparison of them with their high-performance peers in Chinese culture (Morony et al., 2013). For instance, Zhang et al. (2019) meta-analyzed 49 studies and found a negative relationship between mathematics anxiety and math performance and this relationship was stronger for Chinese students than for Western students.

That is, the high school stage is an important period for mainland Chinese students because they must select the category of their future career in terms of their math achievements and might have a higher level of anxiety in mathematics learning. According to China’s education system, general high school students should take the National College Entrance Examination (NCEE), which includes three main subjects (i.e., Chinese, Mathematics, and English) and six additional subjects (i.e., physics, chemistry, biology, history, politics, and geography). Whether or not students choose to take one or more of the subjects of physics, chemistry, or biology during the NCEE determines the coverage of STEM-related majors that they can choose when they enter university. For example, in Shanghai, if students choose to take both physics and chemistry subjects, they will be eligible to apply for most STEM-related majors, while students who choose neither physics nor chemistry will be greatly restricted in their choice of majors in the STEM field. Therefore, the investigations about students who prepare to pursue a STEM major and develop a vocational interest in STEM are concentrated on high school samples. Researchers should develop a reliable attitude scale to measure high school students’ attitudes toward mathematics based on the characteristics of mainland China samples. This would help educators improve their teaching strategies by gaining a better understanding of students’ attitudes toward mathematics.

### 2.2. The Construction of the Survey of Attitudes Toward Mathematics

According to the literature, attitude is a psychological term that is used to describe the degree of an individual’s favor or disfavor of a particular object. Researchers have permitted that one person can simultaneously hold both positive and negative attitudes toward the same object (Wood, 2000). Thus, Carl Gustav Jung defined attitude as a psyche status of acting or reacting in a certain approach and further developed four function types of attitudes: sensing, intuiting, thinking, and feeling (Main, 2004). To be specific, Rosenberg and Hovland (1960) proposed the tri-component of attitude: cognitive, affective, and behavioral. The tri-component structure of attitude has become a popular model of attitude (Breckler, 1984). However, empirical research has confirmed that the distinctions among cognitive, affective, and behavioral intentions are clear and are closely related because cognitive and behavioral components could be the derivative of the affective component (Fazio & Michael, 2003). Therefore, to perfect the structure of attitudes, Fazio and Michael (2003) further developed the intra-attitudinal structure of attitudes by exploring the problem of how an attitude is made. The expectancy–value theory (Atkinson, 1957) is one great effort to understand “how an attitude is made”. According to the expectancy-value theory, the interaction between two factors of expectancies for success and subjective task values contribute to outcomes such as engagement, interest, and achievement. For example, Eccles et al. (1983) developed an expectancy–value model of achievement choice to understand students’ performance and choice in mathematical achievement levels. Here, expectancies are defined as people’s beliefs about their ability to complete a task successfully, while values are expressed as how important, useful, or enjoyable people evaluate a task. The values in the target model include four components of subjective values: attainment, intrinsic, utility, and cost.

Unlike the value components constructed in Eccles et al.’s (1983) model, the component of emotion anxiety should be added to the framework of expectancy–value theory to construct a scale of attitude toward mathematics for Chinese samples in the current study. This is caused by the special situation that the majority of Chinese students have greater self-confidence in learning mathematics but have higher anxiety about mathematics examinations (e.g., Leung, 2006; Morony et al., 2013).

On the other hand, the expectancy–value theory includes intrinsic and extrinsic motivation. This theory integrates these two motivational constructs by emphasizing that both the perceived likelihood of success (expectancy) and the perceived importance, utility, or enjoyment of the task (value) shape students’ motivation. However, the interaction between intrinsic and extrinsic motivation is a complex process (Wigfield & Eccles, 2000). This interaction seems to be more complicated for Chinese students’ value in mathematics. Chinese parents’ education-related expectations of their children were at the top level in PISA 2015, where students were expected to spend more time learning mathematics (OECD, 2016). According to the OECD (2016), 87.6% of Chinese high school students completed their homework after 11 p.m. The expectations from parents or teachers (i.e., extrinsic) and the students themselves (i.e., intrinsic or extrinsic) provide them with value to study hard and perform well. It is necessary to decompose value into two aspects: extrinsic and intrinsic. Therefore, in the current study, we constructed attitudes toward mathematics with two main components of expectancy and value based on the expectancy–value theory. Expectancy includes two sub-components, confidence in mathematics and mathematics anxiety, while value includes two sub-components: intrinsic value and extrinsic value.

First, we defined peoples’ beliefs about their ability to learn mathematics as confidence in mathematics (C). This concept originates from self-efficacy theory (Bandura, 1977), reflecting a person’s confidence in their mastery of mathematical knowledge and skills, as well as the expectation of successfully solving mathematical problems. This self-confidence not only covers positive emotions toward the subject of mathematics itself but also includes optimistic attitudes toward the process of learning mathematics. In educational psychology, self-confidence is considered a key factor in promoting learners to actively participate in learning activities, overcome difficulties, and continue to make progress.

Meanwhile, we defined people’s negative emotions in mathematical learning as mathematics anxiety (A). This sentiment not only pertains to negative feelings towards the subject of mathematics but may also encompass excessive concerns about the difficulties and challenges that may be encountered in the learning process. Mathematics anxiety can stem from a variety of factors, such as an individual’s past learning experiences, self-assessment of mathematical abilities, and fear of failure. In educational psychology, mathematics anxiety is considered a significant barrier affecting students’ motivation to learn, level of engagement, and academic achievement.

On the other hand, we classified values in mathematics into intrinsic value and extrinsic value according to the definition of value in the expectancy–value theory. Intrinsic value, named as enjoyment of mathematics (E), refers to a person’s intrinsic value in mathematics, such as actively enjoying the learning of mathematics. This intrinsic value encourages individuals to engage in learning out of a love for mathematics and enjoyment of the learning process itself, rather than merely pursuing external rewards or outcomes. Enjoying mathematics is usually closely related to individuals’ active participation in mathematical activities, exploratory spirit, and sense of achievement after solving problems. From the perspective of Self-Determination Theory (SDT), enjoying the study of mathematics is seen as an important form of intrinsic value that can promote students’ autonomous learning, sustained engagement, and positive response to challenges. When students feel enjoyment in learning mathematics, they are more likely to exhibit higher learning motivation, better learning outcomes, and a more enduring interest in learning. Therefore, understanding and promoting students’ enjoyment in learning mathematics is significant for stimulating their intrinsic value, improving learning efficiency, and cultivating lifelong learners. Educators should strive to create supportive and challenging learning environments to enhance student’s interest in learning mathematics.

Value of mathematics (V) refers to a person’s extrinsic value in mathematics, such as the usefulness, relevance, and worth of mathematics. This sense of value comes from the recognition of the usefulness, relevance, and value of mathematical knowledge in personal life and society. The value of learning mathematics is not only about an individual’s assessment of the practical application and long-term significance of mathematical knowledge but also includes expectations for external rewards and recognition gained in the process of learning mathematics. Specifically, the value of learning mathematics is usually related to an individual’s understanding of the importance of mathematics in career development, problem-solving ability enhancement, and personal interest satisfaction. This sense of value can stimulate an individual’s extrinsic motivation, that is, to learn mathematics to achieve some external outcome, such as academic grades, social recognition, or career opportunities. At the same time, when an individual recognizes the value of learning mathematics, it may also be transformed into intrinsic motivation, prompting the individual to learn mathematics out of love for the subject itself and a sense of satisfaction. Therefore, when discussing the motivation for learning mathematics, it is crucial to consider the multidimensionality of the value of learning mathematics. This includes assessing how individuals associate mathematical learning with personal goals, social expectations, and future career development, as well as how these factors collectively affect an individual’s mathematical learning behavior and achievements.

## 3. Step I: Creating the CAVE Survey

The research team contains two experts in mathematics education and one expert in educational measurement. The first member is a professor of mathematics education with more than 15 years of math teaching and research experience. The second member is a professor of methodology with a focus on educational research of quantitative methods. The last is a lecturer of mathematics education with more than 10 years of teaching experience. Therefore, this research team has rich teaching and research experience. According to the above description of the construction of the survey of attitudes toward mathematics and the characteristics of Chinese education system, the research team constructed attitudes toward mathematics with four components: confidence (i.e., a person’s belief in one’s ability to learn mathematics successfully), anxiety (i.e., a person’s emotion in mathematics learning), value (i.e., a person’s extrinsic motivation in mathematics learning), and enjoyment (i.e., a person’s extrinsic motivation in mathematics learning). Thus, the instrument testing Chinese high school students’ attitudes toward mathematics was called the CAVE (i.e., confidence, anxiety, value, and enjoyment) survey.

Based on these four components, advice from experts in mathematical education, and the existing instruments (e.g., the FSMAS and ATMI), we first developed a pool of 49 items for measuring students’ attitudes toward mathematics in Chinese. Specifically, items 1, 2, 3, 5, 6, 11, 12, 14, 17, 22, and 23 were adapted from the ATMI, and items 7, 8, 10, 15, 21, 26, and 32 were adapted from the FSMAS. The others were created by authors. Because of the complicated variety of constructions of attitudes toward mathematics from prior studies, the purpose of developing the item pool was to provide references for researchers to select and modify items based on our conceptual framework. Then, each of the 49 items was assigned to one of the four constructs after team discussion. Specifically, one item could be assigned to a specific construct if over two of the team members agreed; otherwise, the team discussed and the experts in mathematical education made the final decision. The survey was translated into an English version, shown in Table 1. Items 30, 31, 33, 35, and 36 are presented in the reversal way of the value of mathematics (i.e., see Table 1). The reversal way of description for items can help educators improve the validity of the survey by reducing careless responses (Huang et al., 2012; Meade & Craig, 2012; Woods, 2006). Finally, we identified 11 items for confidence in mathematics, 12 items for mathematics anxiety, 14 items for value of mathematics, and 12 items for enjoyment of mathematics. The finalized survey is a 7-point Likert scale, where students were asked to rate each item on their level of agreement or disagreement (i.e., 1 = strongly disagree, 2 = disagree, 3 = somewhat disagree, 4 = neither agree nor disagree, 5 = somewhat agree, 6 = agree, 7 = strongly agree). 

## 4. Step II: Pilot Testing of the CAVE Survey

### 4.1. Participants

Convenience sampling was used in this study. The data were collected in fall 2023. The sample for the pilot testing consisted of 740 tenth graders, including 327 males and 413 females. They were from a high school in western China. This is a disadvantaged school. This high school is one of the thousands of county-level high schools in mainland China. They did not receive any incentives during this study. The majority of the participants came from countryside families of Han nationality. The response rate was 94.59% with 700 participants, including 312 males and 388 females. The average math score of participants was 90.05 (*SD* = 15.72). The full score of the collected math test was 150. The scores are participants’ math scores from their high school entrance math examinations. This examination was designed for all middle students who will go to high school in the city of Zunyi. The degree of difficulty is 0.65, which was calculated by the average scores of all participants divided by the full score of 150. Generally, the difficulty of the math test in the high school entrance examination ranges between 0.7 and 0.8.

### 4.2. Analysis

The codes of reversal presented items were transformed (i.e., 1→8, 2→7, 3→6, 4→5, 5→4, 6→3, 7→2, and 8→1). For example, if one student answered item 38 with “1”, we transformed the “1” into “7” (i.e., 8 − 1 = 7). After the pairwise deletion of the missing cases, the normality in the distribution of each item was tested first. Then, an exploratory factor analysis (EFA) was conducted on the remained items to examine whether the data supported the expected dimensionality. The weighted least squares (WLS) method was chosen to estimate and extract factors (Muthén & Muthén, 2010) because WLS is a more appropriate estimator for ordinal data than Maximum Likelihood Estimation (Forero et al., 2009; Olsson, 1979). The “Promax” oblique rotation was used because the initial analysis of the four constructs was inter-correlated (i.e., the most significant correlation is −0.60, as shown in Table 2) and the sample size for this pilot study is sufficient for applying the Promax oblique rotation (Adachi, 2004; Kline, 2002). Here, we applied four constructs in the EFA because the theory suggests extracting four constructs. We decided to delete an item if it met one of the three statistical criteria: (a) the cross-loading was greater than 0.28, (b) the factor loading was greater than 1, or (c) the factor loading was less than 0.32. Then, the EFA procedure was repeated to improve the discrimination validity of the survey. Finally, we used Cronbach’s alpha to check the reliability of each scale. R software version 4.3.1 was utilized to analyze the data.

### 4.3. Results

The results of distribution tests showed that 6 items were non-normally distributed (see Table 1), and thus 43 remaining items were tested with EFA. The initial EFA results indicated that six items met the specified criteria for possible exclusion from the final set of items. One item was cross-loaded on two factors (item 38), and one item was cross-loaded on three factors (item 35). Additionally, four items did not significantly load on any factors (items 17, 30, 37, and 47). According to the experts, “mathematics is boring” (i.e., item 46) should measure the enjoyment of mathematics. However, the EFA results suggested removing this item to mathematics anxiety. Therefore, we decided to exclude item 46 from the CAVE survey. The factor loading results of EFA with 43 items are reported in Table 1. In summary, 13 items were excluded from the item pool.

As a result, 36 items were kept: 11 items were in the scale of confidence, 11 items were in the scale of anxiety, 6 items were in the scale of value, and 8 items were in the scale of enjoyment. Reliabilities of scales were acceptable as they were above or around 0.70 (Blunch, 2008). Reliability coefficients were α = 0.90 (11 items) on the anxiety scale, α = 0.91 (11 items) on the confidence scale, α = 0.71 (8 items) on the enjoyment scale, and α = 0.70 (6 items) to the value scale, respectively. The lower reliabilities of the motivation and the value scales might be caused by the shorter items they had (Charter, 2019; Savalei, 2018).

## 5. Step III: Cross-Validation of the CAVE Survey

### 5.1. Participants

We obtained a sample of 790 students from the same high school in mainland China but offered different classes. The response rate is 98.48% with 778 students, including 333 males and 445 females. The participants took the same math exam (i.e., the total score was 150 and the degree of difficulty was about 0.65) as the pilot sample, and the average math score was 107.80 (*SD* = 13.59).

### 5.2. Analysis

To examine if the survey structure fits the theory of attitudes toward mathematics, a confirmatory factor analysis (CFA) was conducted on the main data with 778 responses by using R with the WLS estimation method. Based on the results of the pilot study, the structure of the tested model consists of four factors with 8 items on the enjoyment scale, 11 items on the anxiety scale, 6 items on the value scale, and 11 items on the confidence scale. It is inappropriate to use chi-square statistics to evaluate the overall fit of the specified model because chi-square statistics show oversensitivity to the large sample size (Flora & Curran, 2004). Thus, in an analogous-to-the-pilot data evaluation, root mean square error of approximation (RMSEA), standardized root mean square residual (SRMR), comparative fit index (CFI), and Tucker–Lewis index (TLI) were used to evaluate the fit of the specified model. For indicating an adequate model fit, CFI and TLI should be above 0.90, and RMSEA and SRMR should be under 0.08; for an excellent fit, CFI is required to be above 0.95, and RMSEA and RMR should be under 0.05 (Hooper et al., 2008). The evaluation of the tested model took the following steps: (a) examine the specifications of parameters, (b) examine the parameter estimates, (c) evaluate the goodness of fit indices, and (d) investigate the ill parameters according to the modification index. The chi-square difference test was used to test the differences between the tested and the revised models.

### 5.3. Results

**Figure 1:**
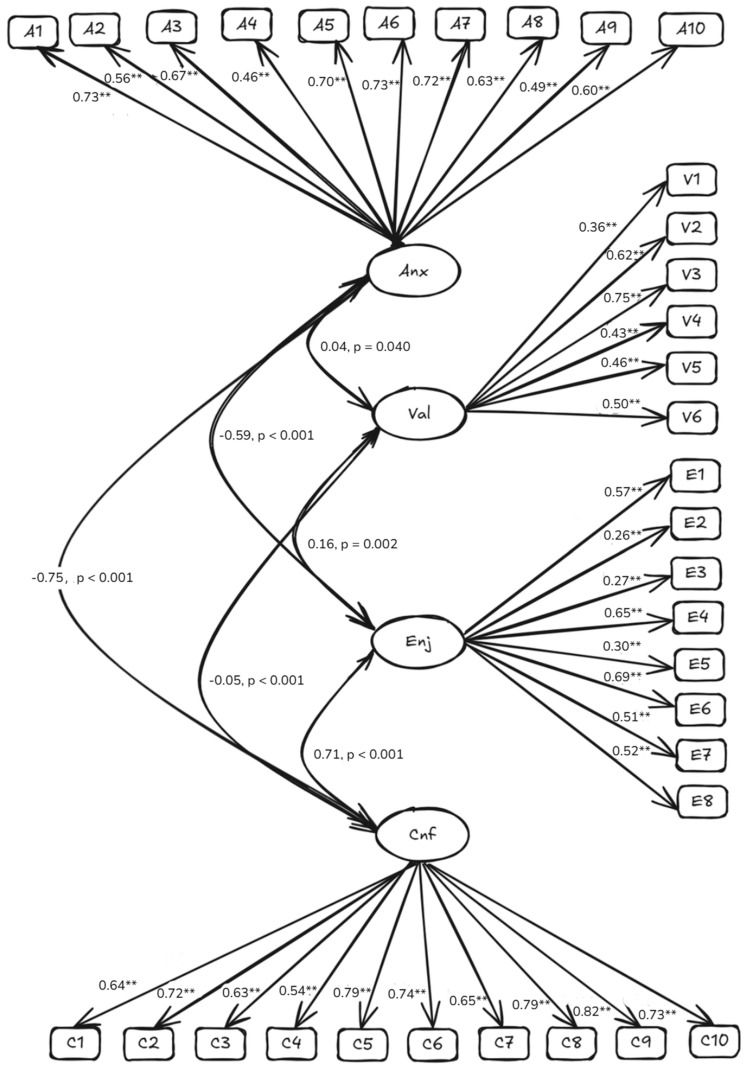
Standardized revised CFA model. Note. Cnf: confidence; Anx: anxiety; Val: value; Enj: enjoyment. ** *p* < 0.001.

The initial CFA results of the tested model with no covariance among error terms showed that the tested model was supported. The z-statistics of factor loadings ranged from 2.45 to 21.86, meaning that all items were loaded on the specified factors statistically significantly (i.e., greater than *z* = 1.96; Hatcher, 1994). According to the model fit index, CFI was 0.88, TLI was 0.87, RMSEA was 0.06, and SRMR was 0.05. Both CFI and TLI were larger than 0.85 but lower than 0.90, and RMSEA and SRMR were lower than 0.06, meaning that the data did fit the tested model well, but the tested model could have better fit with additional improvement. To improve the model fits, the modification index recommended releasing the correlated error terms between items 7 and 8, and between items 15 and 16. We found the sentences of items 7 and 8, as well as items 15 and 16 (see Table 1) have similar meanings. Because item 7 had a lower loading value on anxiety than item 8, as well as item 16 had a lower loading value on confidence than item 15, we removed items 7 and 16 from the instrument. After the changes made for indicator structures, the model fit improved: CFI increased to 0.91, TLI improved to 0.90, and RMSEA decreased to 0.05. The results of the chi-square difference test show that the revised model does fit better than the model with independent errors, χ^2^(67) = 238.22, *p* < 0.001. The standardized solution of the revised model is presented in Figure 1. We found that the value had significantly weak relationship with confidence (*r* = −0.05, *p* < 0.001), anxiety (*r* = 0.04, *p* = 0.040), and enjoyment (*r* = 0.16, *p* = 0.002), respectively. Also, anxiety had significantly negative relationship with confidence (*r* = 0.75, *p* < 0.001) and enjoyment (*r* = −0.59, *p* < 0.001); confidence had a significantly strong relationship with enjoyment (*r* = 0.71, *p* < 0.001). Finally, the reliability coefficients became α = 0.90 (10 items) to the anxiety scale, and α = 0.90 (10 items) to the confidence scale. Interestingly, the reliability of the final survey was α = 0.65, indicating the low correlations between some of the scales.

## 6. Step IV: Another Evidence for the Validity of the Survey

### 6.1. Analysis

Based on the Standards for Educational and Psychological Testing (AERA et al., 2014), we further checked the validity of the new survey by examining the impact of attitude on math performance. Cluster sampling was used at this step. The sample consisted of 1478 participants from the same school, including 645 males and 833 females. The average math score of the participants was 99.57 (*SD* = 17.00). The full score was 150, and the degree of difficulty was 0.65. The scores were participants’ math performance from the high school entrance examination. The multiple regression analysis was utilized to determine the positive or negative effects of the four factors (i.e., confidence, anxiety, value, and enjoyment) on mathematics performance, as well as how much of the variance in mathematics scores could be explained by the four factors. Note that we computed the average rated score of each measured factor for each student as the raw data with each tested factor (i.e., confidence, anxiety, value, and enjoyment), and treated these factors as continuous variables. Preliminary analyses were conducted to ensure there is no violation of the assumptions of normality and multicollinearity.

### 6.2. Results

Table 3 shows the results of the multiple regression analysis. The results indicated an overall significant effect of attitudes on students’ mathematics performance, *F* (4, 1473) = 50.10, *p* < 0.01, adjusted *R*^2^ = 0.12. “Enjoyment of mathematics” (i.e., *t* = 2.11, *p* < 0.05) and “confidence in mathematics” (i.e., *t* = 3.81, *p* < 0.01) have a positive significant effect on students’ mathematics performance. “Mathematics anxiety” impacts students’ mathematics performance negatively, *t* = −8.19, *p* < 0.01. However, the “value of mathematics” has on effect on students’ mathematics performance, which is different from other studies (Wigfield & Cambria, 2010; Wigfield & Eccles, 2000). This finding indicates that the characteristics of mainland Chinese students are different from those in other countries. However, it is necessary to recheck the relationship of the value of mathematics and math performance in future studies by using different samples from mainland China.

## 7. Discussion

This study was designed to develop a valid and reliable survey of attitudes toward mathematics for high school students in mainland China. The finalized survey was identified to be composed of four scales: confidence in mathematics (10 items), mathematics anxiety (10 items), value of mathematics (6 items), and enjoyment of mathematics (8 items). The results of factor analysis and acceptable internal reliabilities of scales confirmed that the developed four-scale is an appropriate tool to examine students’ attitudes toward mathematics. Moreover, because only 34 items are included, the estimated time for students to complete the assessment is acceptable, ranging from 10 to 20 min.

### 7.1. The Theoretical Framework and Specific Components of the Developed Survey

In the created survey, we specifically designed positively and negatively described items to measure the same dimension of the value of the mathematics scale. We did this because (a) it can help educators to measure students’ attitudes more accurately, and (b) previous scales (e.g., ATMI) used the positively and negatively described items together to ensure their high reliabilities. In fact, according to the Chinese educational system, it is common for students to have complicated opinions about the value of mathematics. However, the situation is more complex than we assumed, where half of the negatively worded items were non-normal distributed with more agreements on the negative value of mathematics (i.e., see items 31, 33, and 36 in Table 1). Meanwhile, the other negatively worded items tend to cross-load with the mathematics anxiety and enjoyment of mathematics (e.g., see items 30 and 35 in Table 1). The remaining positively worded items all emphasize the external value of mathematics, for example, “My parents especially hope that I can learn mathematics well”, “I hope to learn mathematics well, so that I can get praise from teachers”, and “Mathematics is very important for the college entrance examination. I hope to learn mathematics well”. That is, mainland Chinese students’ judgments on the value of mathematics mainly come from the external world, and lack of their internal feelings on the value of mathematics. On the other hand, the results show the positively and negatively worded items should measure different dimensions. It is consistent with the study of Lim and Chapman (2013). This finding indicates a need to subdivide positively and negatively worded items to make the constructs of any instruments more representative.

### 7.2. The Psychometric Properties of the Developed Survey

Another interesting finding is that the final survey showed slightly low internal reliability (i.e., α = 0.65), whereas the internal reliability was acceptable for each scale (i.e., α was above 0.70). First, this might be related to the low correlations between some of the scales. The value had significantly weak relationships with confidence (*r* = −0.05, *p* < 0.001), anxiety (*r* = 0.04, *p* < 0.05), and enjoyment (*r* = 0.16, *p* < 0.01), respectively. However, the weak relationships rarely happened in other studies with a higher internal consistency of their overall scale, in comparison to their subscales. That is, most of the absolute values of correlation coefficients between factors were more than 0.30. For example, in the study of Lim and Chapman (2013), the correlation coefficients between anxiety and enjoyment, emotion, confidence, and value were −0.59, −0.56, −0.90, and −0.38, respectively. The different results again demonstrated the different characteristics between Taiwanese and mainland Chinese samples, indicating that more studies on mainland Chinese samples are necessary. Second, the modest internal reliability may be caused by the fact that items were chose to represent the conceptual breadth within the four-dimension construct rather than maximize internal consistency (Ryff & Keyes, 1995). The current survey includes two main components: expectancy (confidence and anxiety) and value (enjoyment and value). Each component includes two opposite aspects. This is the characteristics of the CAVE survey. This construct could contribute to the low internal consistency. Meanwhile, this psychometric characteristic is needed to be examined in the future studies by using Chinese sample because of the negative relationship between value and confidence (i.e., *r* = −0.05) in this study.

Furthermore, the specifically negative relationship between value and confidence (i.e., *r* = −0.05) in the current study was inconsistent with prior studies, where only positive relationships were supported. For example, Lin and Huang (2016) found the correlations between value and confidence were *r* = 0.33 for one sample and *r* = 0.46 for another sample, and the relationship between value and confidence reported in Ramazan and Seher (2015) was also positive with *r* = 0.51. The negative relationship between value and confidence suggests that the sample of Chinese students with high confidence in learning mathematics may not think that mathematics is valuable for them. The same finding was found in the results of multiple regression analysis in the present study, indicating that mainland Chinese students’ hard work and high performance are not caused by their internal recognition of mathematics value. These echoed the statement that the Asian students’ mathematical values were poor and below the international average, although their mathematics scores were at the top level of the TIMSS-R (TIMSS 1999; Mullis et al., 2000).

As Leung (2006) stated, Asian students’ attitudes toward mathematics may have been deeply affected by their culture. The poor mathematical values of Chinese students might be caused by the features of the Chinese educational system. As the teacher-central teaching style is commonly used in mainland China, students are pushed to learn mathematics in their daily learning by teachers or parents. More specially, with the application of the teacher-central teaching strategy, teachers would like to emphasize the understanding of procedural and conceptual mathematical knowledge by employing traditional rigid practice (e.g., An et al., 2004; Cai & Ding, 2017; Wang & Lin, 2005). This teaching style might affect students’ realization of the value of mathematics during their learning process. Thus, regardless of the necessity of mathematics for the college entrance examination, students may not agree that mathematics is valuable for their lives and have low enthusiasm to pursue mathematics as a major in the future. On the other hand, the non-significant positive correlation between anxiety and value (*r* = 0.04) also demonstrated the difference between students from mainland China and other regions (e.g., Lim & Chapman, 2013; Wigfield & Meece, 1988). For example, the relationship between anxiety and value was negative (*r* = −0.38) in the study of Lim and Chapman (2013). This shows that students in mainland China remain anxious about mathematics, though they have high confidence in learning mathematics and could achieve high performance in mathematics.

Most importantly, although the developed instrument does not directly measure students’ knowledge of STEM, the practical application of attitudes toward mathematics in terms of the four scales is important for STEM education. According to Wang (2013), for students, the factors of attitudes toward mathematics, mathematical self-efficacy beliefs, intent to pursue the STEM field, and aspiration to earn a STEM graduate degree have a significant relationship with STEM entrance. She further highlighted the importance of cultivating students’ positive attitudes toward mathematics from early on. Meanwhile, Sahin et al. (2018) found that students with higher math and science self-efficacy were more likely to select a STEM major in college, and Bicer et al. (2020a) found that students with higher math self-efficacy were more likely to come up with creative ideas in STEM-related problems. Therefore, the current attitudes toward the mathematics survey are one part of the authors’ efforts in the field of STEM education, including interventions to improve students’ interest in STEM majors and pursue STEM careers in the future.

### 7.3. Limitations and Future Directions

Although the current study developed a valid and reliable measure of high school students’ attitudes toward mathematics in Chinese, it is not free of limitations. First, the appropriate number of items could be added to the enjoyment and the value scales. The reliabilities of the enjoyment (i.e., α = 0.71 with eight items) and value (i.e., α = 0.70 with six items) scales were relatively lower and need to be improved. The lower reliabilities might be caused by the smaller number of items they had (Charter, 2019; Savalei, 2018; Yang & Green, 2015). If we could include more items to measure enjoyment and value, the internal consistencies of the two scales would be increased. On the other hand, completing the survey within an appropriate time is also important. Bradbury (2016) claimed that students can pay attention up to 20 min, and the number of items on the survey cannot be too large. However, the typical appropriate number of items, changing in terms of the length and content of each item, is still unknown. Thus, more studies are needed to confirm the acceptable number of items for typical surveys.

Second, it is necessary to include more aspects of attitudes toward mathematics depending on the samples. The appropriate instrument must cover important dimensions of attitudes toward mathematics. According to the theoretical construction and findings of the current study, it is better to subdivide the positively and negatively worded items within one scale as different dimensions. The subdivision with scales of confidence, anxiety, value, and enjoyment could provide a clearer theoretical structure with accuracy. On the other hand, we only selected tenth graders from western China as samples to develop the survey. However, the construct of the survey may be varied with a change of samples. Hence, further investigations using samples from different high school grade levels (i.e., grades 11 and 12) and other regions of mainland China are needed. Moreover, future studies could administer this new survey and another survey known in the field of mathematics education together to provide validity evidence about whether this new survey is measuring students’ attitudes towards mathematics similar to another survey. Also, the negative relationship between value and confidence in this study, which is different from other studies (Adelson & McCoach, 2011; Shone et al., 2024), is worth looking into by possibly following up with qualitative studies in the future.

Third, we did not explore the relationships among attitudes toward mathematics, STEM interest, and STEM achievement because of the topic of the current study. It is explained in the Introduction that mathematical literacy is the foundation for the development of STEM education. Because success or failure in mathematical achievement is greatly determined by attitudes, an investigation of the track-change of students’ attitudes toward mathematics in the high school stage may be meaningful for students’ career selection in STEM. Hence, attitudes toward mathematics may have significant positive relationships with STEM interest, STEM career, STEM literacy, and integrated STEM education. Future studies are advised to confirm these relationships and create a new theoretical model including these parameters.

## 8. Conclusions

This study developed the Chinese version of a survey of attitudes toward mathematics for high school students based on prior studies and the characteristics of the educational system in mainland China. The finalized survey includes four scales: confidence (i.e., α = 0.90 with 10 items), anxiety (i.e., α = 0.90 with 10 items), value (i.e., α = 0.70 with 6 items), and enjoyment (i.e., α = 0.71 with 8 items). The current study can contribute to the development of mathematics education research in terms of both theoretical and practical perspectives. After the development of the survey, researchers can (a) develop new surveys for attitudes toward mathematics based on this survey, and (b) revise it based on their research subjects or fields.

## Figures and Tables

**Table 1:** Factor loading structure of 49 items.

Item	Distribution	Initial Factor ^a^	Factor				Final Factor ^b^
			A	C	E	V	
1. Mathematics doesn’t frighten me at all	Normal	C	−0.26	**0.49**	0.03	0.00	C
2. I am sure of myself when I do math	Normal	C	−0.16	**0.60**	0.09	−0.01	C
3. I have the ability to solve math problems without too much effort	Normal	C	0.03	**0.75**	0.00	−0.07	C
4. I think I can do well in learning the content of each mathematics class	Normal	C	0.06	**0.53**	0.14	−0.07	C
5. In the process of learning mathematics, I feel very relaxed	Normal	C	0.03	**0.71**	0.11	−0.06	C
6. I think I’m good at solving math problems	Normal	C	0.03	**0.72**	0.07	−0.02	C
7. I never get nervous in math exams	Normal	C	−0.10	**0.56**	−0.14	0.08	−
8. I usually feel relaxed in math exams	Normal	C	−0.03	**0.64**	−0.12	0.05	C
9. I usually don’t worry about solving math problems because I believe I have the ability	Normal	C	−0.04	**0.69**	0.04	−0.02	C
10. I usually feel relaxed in mathematics course	Normal	C	−0.01	**0.54**	0.12	−0.08	C
11. I’m not afraid of mathematics at all	Normal	C	−0.20	**0.48**	0.03	0.06	C
12. Mathematics is one of my most feared subjects	Normal	A	**0.54**	−0.13	0.04	−0.05	A
13. When I use mathematics in my daily life, my brain is blank	Normal	A	**0.57**	−0.12	0.02	−0.10	A
14. When I study mathematics, I always feel nervous	Normal	A	**0.69**	−0.14	0.19	−0.07	A
15. Mathematics makes me uncomfortable	Normal	A	**0.74**	0.11	−0.11	−0.06	A
16. When I heard the word math, I had a feeling of dislike	Normal	A	**0.67**	0.13	−0.16	−0.08	−
17. In mathematics class, I am often confused	Normal	A	0.31	−0.18	0.10	0.04	−
18. Mathematics makes me feel uncomfortable, irritable and anxious	Normal	A	**0.57**	−0.05	−0.08	−0.01	A
19. I often feel frustrated when I try to solve difficult math problems	Normal	A	**0.46**	−0.11	0.11	0.00	A
20. I’m afraid of math exams	Normal	A	**0.69**	−0.14	0.16	−0.04	A
21. Mathematics usually makes me uncomfortable and nervous	Normal	A	**0.77**	−0.02	0.02	0.00	A
22. Math has been my worst subject	Normal	A	**0.51**	−0.20	−0.02	0.06	A
23. Mathematics makes me feel uneasy or confused	Normal	A	**0.53**	−0.17	0.00	0.10	A
24. My parents especially hope that I can learn mathematics well	Normal	V	−0.03	−0.12	−0.03	**0.55**	V
25. I hope to learn mathematics well, so that I can get the praise from teachers	Normal	V	0.14	0.07	0.03	**0.51**	V
26. I know that if I learn math well, it is easier for me to find a job in the future	Normal	V	−0.07	−0.02	−0.05	**0.72**	V
27. Mathematics is very important for college entrance examination. I hope to learn mathematics well	Non-normal	V	−	−	−	−	−
28. Doing well in math can help me to find a better job in the future	Normal	V	−0.02	−0.08	0.16	**0.37**	V
29. I’ll need a good understanding of math for my future work	Normal	V	−0.13	0.04	−0.09	**0.66**	V
30. * It’s very difficult to get the teacher’s approval and praise if you can’t do well in mathematics	Normal	V	0.28	0.07	−0.07	0.28	−
31. * Doing well in math is not important for my future	Non-normal	V	−	−	−	−	−
32. My teacher wants me to do well in mathematics	Normal	V	−0.03	−0.07	0.03	**0.44**	V
33. * For human beings, other subjects are more important than mathematics	Non-normal	V	−	−	−	−	−
34. Mathematics has made an important contribution to the progress of civilization	Non-normal	V	−	−	−	−	−
35. * Math will not be important to me in my life’s work	Normal	V	−0.34	−0.28	0.28	0.04	−
36. * I will study liberal arts in the future, and I don’t need to be proficient in mathematics	Non-normal	V	−	−	−	−	−
37. I would like to study science (Physics, Chemistry, and Biology), Technology, Engineering, Mathematics, and other relative subjects after I enter university, as my major.	Normal	V	0.10	0.16	0.26	0.17	−
38. * Math is not a very interesting subject	Normal	E	0.33	0.11	0.34	0.10	−
39. Mathematics is very worth to learn, and I want to study mathematics as my profession	Normal	E	0.09	0.25	**0.44**	0.07	E
40. * I’m not passively learning mathematics	Normal	E	0.02	0.20	**0.47**	−0.10	E
41. I am interested in acquiring further knowledge of mathematics	Normal	E	0.10	0.10	**0.64**	−0.11	E
42. Mathematics helps me to develop my mind and helps me to think	Normal	E	0.02	0.07	**−0.38**	−0.08	E
43. I like trying to solve new problems in mathematics	Normal	E	−0.02	0.27	**0.44**	−0.09	E
44. Understanding mathematical concepts made me exciting	Normal	E	0.00	−0.13	**0.49**	0.06	E
45. Mathematics is pleasant and exciting to me	Normal	E	−0.09	0.13	**0.60**	−0.07	E
46. * Mathematics is boring	Normal	E	**0.42**	0.13	−0.23	0.00	−
47. I hope that I can learn mathematics well and solve the important mathematical problems in the future	Normal	E	0.16	0.24	0.28	0.28	−
48. I have a sense of achievement in doing well in math	Non-normal	E	−	−	−	−	−
49. I’m willing to study mathematics outside the college entrance examination	Normal	E	−0.05	0.19	**0.41**	0.17	E

Note. C: confidence; A: anxiety; V: value; E: enjoyment. Factor loading coefficients that are considered as “could be chosen” are shown in bold. ^a^ The initial factor that is supposed to reflect the item. ^b^ The final factor that is supposed to reflect the item. * means that the description is a reversal.

**Table 2:** Factor correlation matrix.

Factor	Confidence	Anxiety	Value	Enjoyment
Confidence	1.00			
Anxiety	−0.60 **	1.00		
Value	−0.14 **	0.08 *	1.00	
Enjoyment	0.40 **	−0.51 **	0.19 **	1.00

* *p* < 0.05; ** *p* < 0.01.

**Table 3:** Multiple regression analysis.

	B	Std. Error	β	*t* Value	*p* Value
Intercept	103.90	3.58		29.05	<0.01
Enjoyment	1.07	0.51	0.07	2.11	0.03
Anxiety	−3.97	0.49	−0.24	−8.19	<0.01
Value	−0.38	0.43	−0.02	−0.89	0.37
Confidence	1.92	0.50	0.11	3.81	<0.01
Adjusted *R*^2^	0.12				
*F*-statistic	50.10				

## Data Availability

The data presented in this study are available on reasonable request from corresponding author. The data are not publicly available due to ethical considerations.
